# Dependence and Homeostasis of Membrane Impedance on Cell Morphology in Cultured Hippocampal Neurons

**DOI:** 10.1038/s41598-018-28232-0

**Published:** 2018-07-02

**Authors:** Ryosuke Matsumura, Hideaki Yamamoto, Takeshi Hayakawa, Shutaro Katsurabayashi, Michio Niwano, Ayumi Hirano-Iwata

**Affiliations:** 1 Research Institute of Electrical Communication, Tohoku University, 2-1-1 Katahira, Aoba-ku, Sendai, 980-8577 Japan; 2 Frontier Research Institute for Interdisciplinary Sciences, Tohoku University, 6-3 Aramaki-aza Aoba, Aoba-ku, Sendai, 980-8578 Japan; 3 Faculty of Pharmaceutical Sciences, Fukuoka University, 8-19-1 Nanakuma, Jonan-ku, Fukuoka, 814-0180 Japan; 4 WPI-Advanced Institute for Materials Research (WPI-AIMR), Tohoku University, 2-1-1 Katahira, Aoba-ku, Sendai, 980-8577 Japan; 5 Present Address: WPI-AIMR, Tohoku University, Sendai, Japan; 6 Present Address: Kansei Fukushi Research Institute, Tohoku Fukushi University, 6-149-1 Kunimigaoka, Aoba-ku, Sendai, 989-3201 Japan

## Abstract

The electrical impedance of cell membranes is important for excitable cells, such as neurons, because it strongly influences the amount of membrane potential change upon a flow of ionic current across the membrane. Here, we report on an investigation of how neuronal morphology affects membrane impedance of cultured hippocampal neurons. Microfabricated substrates with patterned scaffolding molecules were used to restrict the neurite growth of hippocampal neurons, and the impedance was measured via whole-cell patch-clamp recording under the inhibition of voltage-dependent ion channels. Membrane impedance was found to depend inversely on the dendrite length and soma area, as would be expected from the fact that its electrical property is equivalent to a parallel RC circuit. Moreover, we found that in biological neurons, the membrane impedance is homeostatically regulated to impede changes in the membrane area. The findings provide direct evidence on cell-autonomous regulation of neuronal impedance and pave the way towards elucidating the mechanism responsible for the resilience of biological neuronal networks.

## Introduction

In the central nervous system, response of neurons to an oscillatory input varies depending on the signal frequency^1–7^. This electrical property is governed by the impedance spectra of the neuronal cell membrane and plays a crucial role in determining the preference of neurons and their networks to operate at a certain frequency. It is also related to the generation of field potential rhythms that are involved in the neural information processing^7,8^.

The frequency characteristics of neurons are determined by the interaction of active and passive properties within the plasma membrane^3,9^. For the former, membrane currents mediated by the hyperpolarization-activated cyclic nucleotide-gated (HCN) channels and the M-type K^+^ channels have been identified as the molecular mechanism that endows frequency selectivity to neurons^2,9–15^. In the stellate cells in the entorhinal cortex, for example, the currents cause the neurons to respond largest to oscillating inputs around 5–15 Hz, which correspond to the theta-band range^16–18^. In pyramidal neurons, which are the major neuron in the hippocampus and the neocortex, the persistent Na^+^ current counteracts with M-current, and membrane impedance *Z*_m_ exhibit either a less distinct resonance at low frequencies (1–5 Hz) or no resonance at resting potentials^10,15–17,19^.

The passive property of the membrane is defined by the leak conductance and the lipid bilayer acting as the resistive and capacitive components, respectively. Considering that the passive property is equivalently modelled as a parallel RC circuit, perturbation in cell morphology and membrane area would pose substantial change in neuronal impedance. Despite this effect, neuronal morphology fluctuates during development and in mature brains via, e.g., formation/elimination of synaptic spines^20^ and dendritic arbors^21^. Irregularity in neuronal size and morphology have been reported in patients and in animal models of several neurological disorders, including autism and epilepsy^22–24^. However, it is still unclear how cell morphology affects *Z*_m_, which is partly due to the lack of an experimental platform that permits direct investigation of this effect.

Here, we report on an experimental study of the effect of cell morphology on impedance spectra, with a particular focus on the passive membrane property. This work is grounded on the surface engineering technique that permits the extrinsic control of the neurite outgrowth of cultured neurons at a single cell resolution^25–32^. Membrane impedance, as measured by whole-cell patch-clamp recording, is compared between neurons with and without growth control at identical days *in vitro* (DIV). Conductance-based simulations were used to theoretically examine the underlying biophysical mechanisms.

## Results

### Neuronal micropatterning

**Figure 1:**
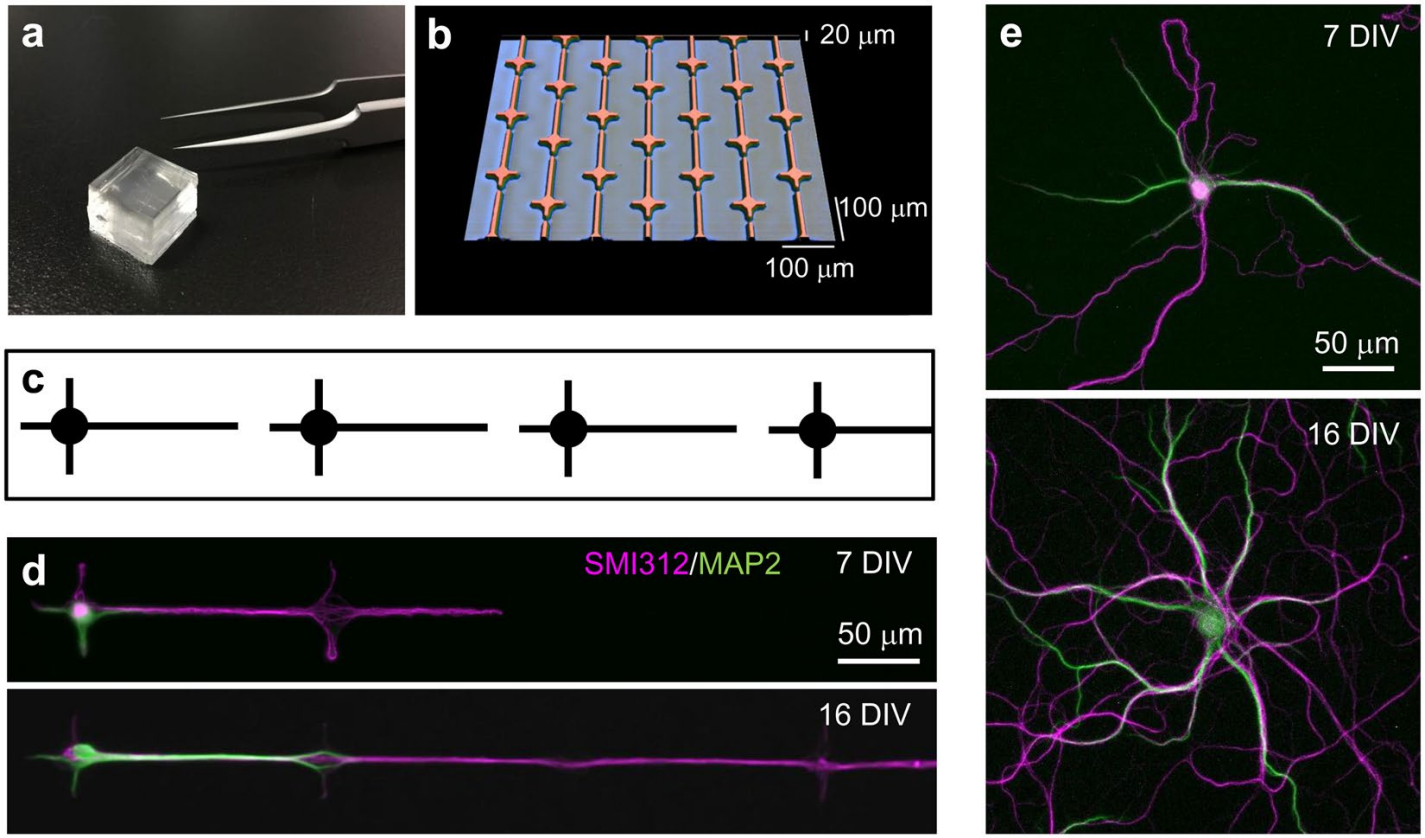
Photograph (**a**) and 3D confocal micrograph (**b**) of a PDMS stamp. (**c**) Schematic illustration of the micropattern geometry. Patterned (**d**) and unpatterned (**e**) neurons at 7 and 16 DIV stained with axonal (SMI312) and somatodendritic (MAP2) markers.

Glass coverslips for culturing neurons were fabricated by microcontact printing^29–32^. Briefly, cleaned glass coverslips were first coated with agarose to produce a cytophobic surface, and a cell-adhesive ink, consisting of an extracellular matrix protein gel and poly-lysine, was stamped using a microstructured polydimethylsiloxane (PDMS) stamp (Fig. 1a,b). Dissociated rat hippocampal neurons were then plated on and cultured on the patterned glass coverslip. Approximately 90% of the cells in the hippocampal cultures are the pyramidal neurons^33^. As illustrated in Fig. 1c, micropatterns consisted of a circular island (25 μm in diameter), from which four lines emerged forming a cross, one 100 μm long and three 20 μm long, and they were aligned with a minor gap of 10 μm that axons could surpass^31^. At 7 and 16 DIV, the cell morphology and *Z*_m_ were characterized by immunostaining and whole-cell patch-clamp recordings, respectively. Neurons that were grown on a conventional poly-lysine coated coverslip for identical periods were prepared as a control sample, and the effect of restricting dendrite length and arborization was compared between the patterned and unpatterned neurons.

Figure 1d shows the development of rat hippocampal neurons on micropatterned substrates. Cell bodies preferentially settled on the circular island, and neurites grew on the line patterns. As the cultures matured, axons became elongated onto distant patterns. In contrast, most of the dendrites that grew on the short pathways did not grow any further. In the unpatterned neurons, dendrites elongated freely without restrictions (Fig. 1e).

**Figure 2:**
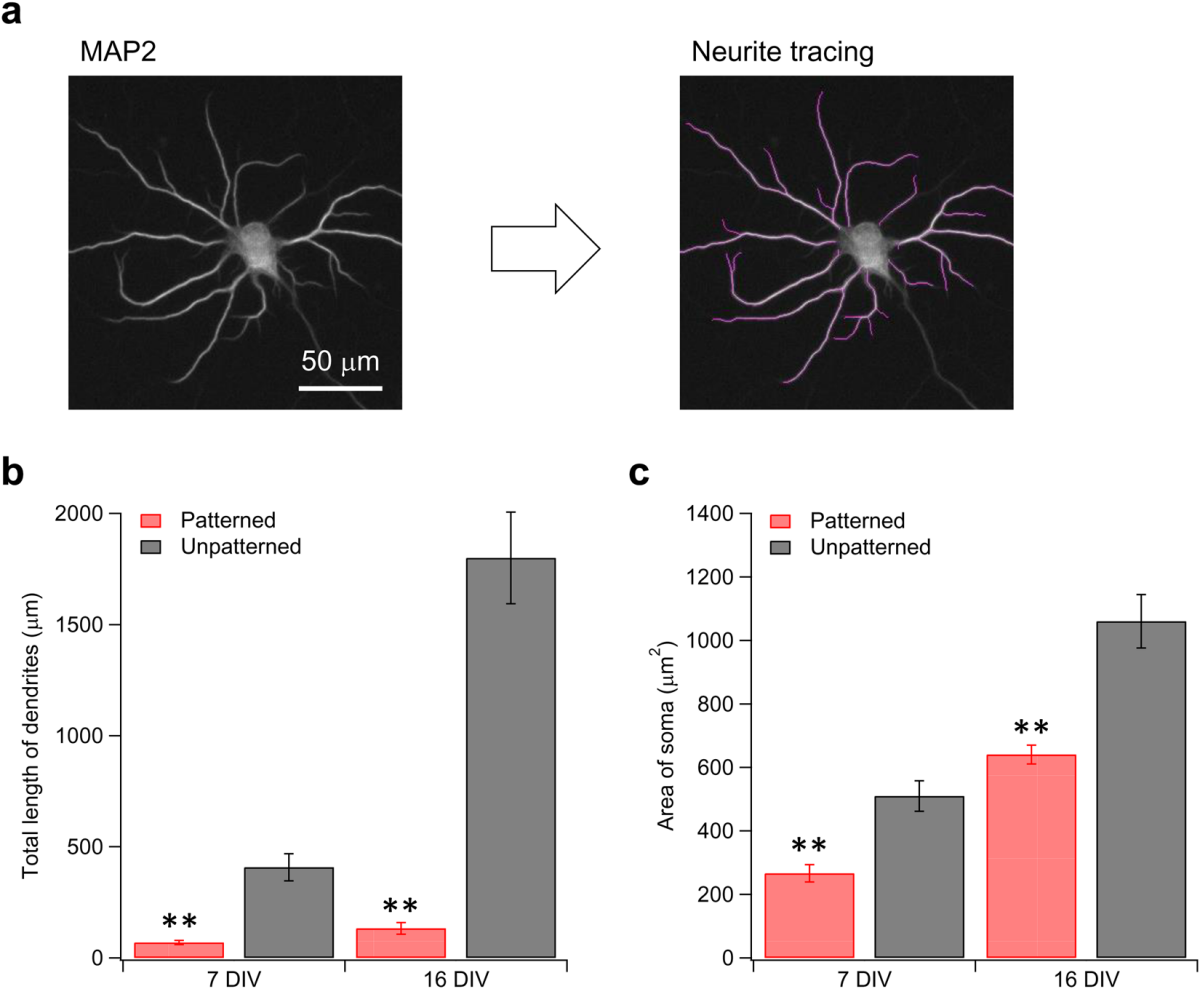
(**a**) Micrograph of a typical unpatterned neuron at 16 DIV stained with MAP2. Pink lines are the tracings of neurites. (**b**) Total length of dendrites obtained from patterned (*n* = 7, red) and unpatterned (*n* = 7, black) neurons at 7 and 16 DIV. (**c**) Area of soma obtained from and unpatterned neurons at 7 and 16 DIV. Error bars indicate SEM. **p < 0.01 (one-sided *t*-test).

Figure 2 summarizes the soma area and the total dendritic length of each neuron, which were evaluated from fluorescence micrographs of neurons stained with a somatodendritic marker, MAP2. A significant difference in both parameters was confirmed between the patterned and unpatterned neurons. Thus, the morphological confinement via micropatterning effectively reduced the total surface area of the cell membrane. Note that axons have less influence on *Z*_m_ due to their high axial resistance (see the Simulation results. section).

### Electrophysiological measurements

**Figure 3:**
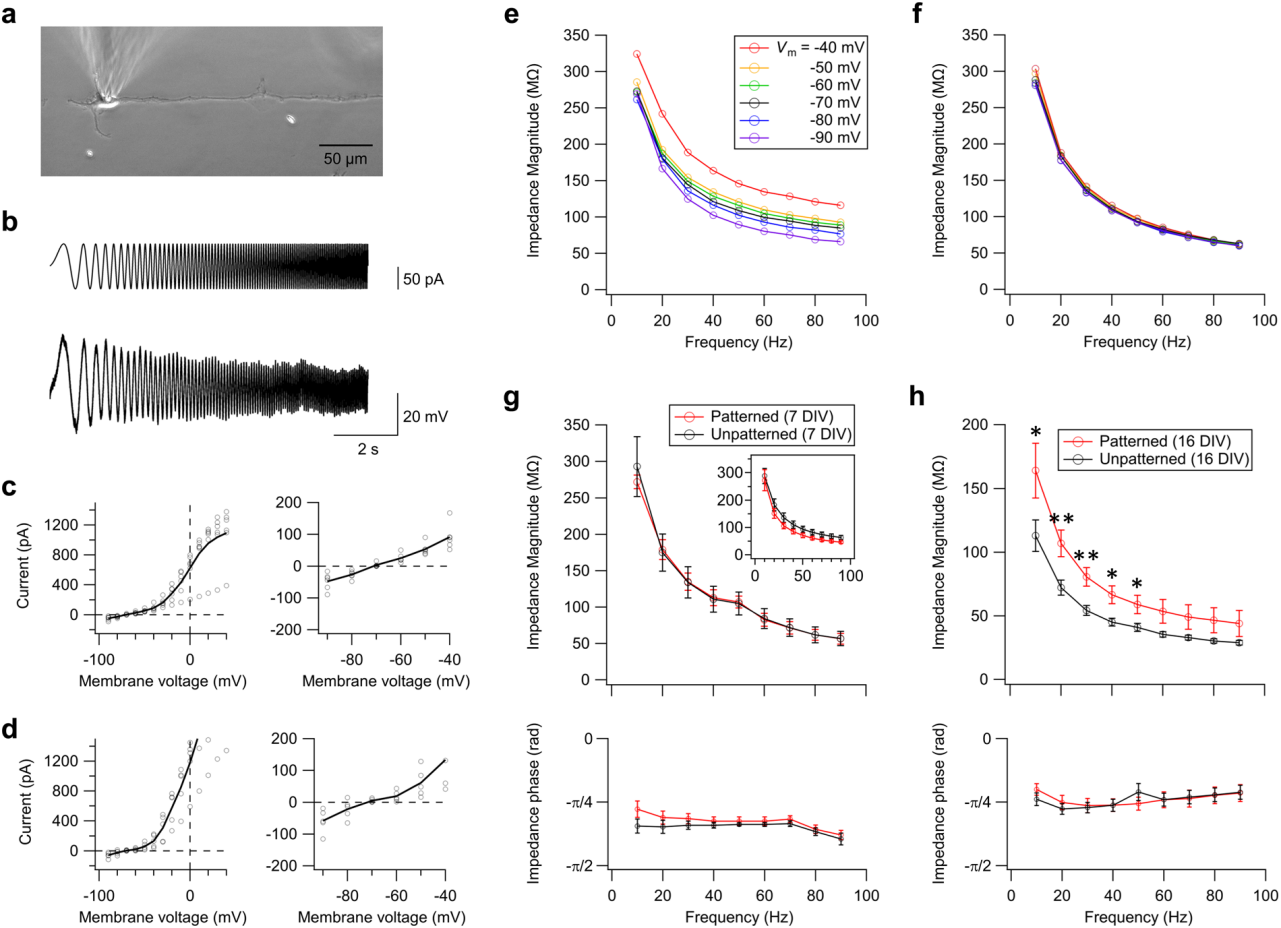
(**a**) Micrograph of a patterned neuron at 7 DIV during a patch-clamp recording. (**b**) Typical traces of the applied chirp current (top) and the voltage response (bottom). Only the first 10 s are shown. (c,d) Current-voltage relationships of (**c**) unpatterned (*n* = 6) and (**d**) patterned (*n* = 5) neurons at 7 DIV. The open circles and the solid line represent individual measurements and the mean, respectively. Magnification of the voltage range of −90 mV to −40 mV is shown in the right panels. (**e**) Effect of membrane potential (*V*_m_) on the impedance magnitude profile (*n* = 6; 7 DIV; unpatterned). Error bars not shown to aid visualization. (**f**) Impedance spectra at various *V*_m_ obtained in the presence of TEA (*n* = 5; 7 DIV; unpatterned). (**g**,**h**) Impedance spectra of patterned (*n* = 5, red) and unpatterned (*n* = 5, black) neurons at (**g**) 7 DIV and (**h**) 16 DIV (*V*_m_ = −70 mV). Upper and lower panels show the impedance magnitude and phase profiles, respectively. Inset in (**g**) shows the impedance magnitude profile of patterned (*n* = 4) and unpatterned (*n* = 5) neurons recorded in the presence of TEA. Error bars indicate SEM. *p < 0.05; **p < 0.01 (one-sided *t*-test).

Next, in order to evaluate the effect of morphological confinement on *Z*_m_ and its dependence on the age of the culture, we performed patch-clamp recordings on patterned and unpatterned neurons (Fig. 3a). In each recording, a chirp current that consisted of a sinusoidal wave with a constant amplitude (50 pA), and a time-varying frequency (0.1–100 Hz) was applied under the current-clamp mode with a current injection to maintain the membrane potential (*V*_m_) at −70 mV. A typical voltage response is shown in Fig. 3b, in which the amplitude decreases with increasing frequency. The frequency response curve was then obtained by calculating the ratio of the Fourier transform of the voltage response to that of the input current. Tetrodotoxin (TTX; 1 μM), a voltage-gated Na^+^ channel blocker, and 6-cyano-7-nitroquinoxaline-2,3-dione (CNQX; 10 μM), an AMPA-type glutamatergic receptor blocker, were added to the external solution during all recordings to suppress the generation of action potentials and synaptic transmissions, respectively. Addition of TTX has been reported to have minor influence on *Z*_m_ in the absence of spikes^15^. CNQX is also expected to have little influence on *Z*_m_, because its target channel is a ligand-gated channel that is usually closed unless glutamate is bound to it.

In order to verify that the membrane property is linear and is dominated by the passive mechanisms near *V*_m_ = −70 mV, we first analysed the current-voltage relationship of hippocampal neurons in the presence of TTX and CNQX. For both the unpatterned (Fig. 3c) and patterned (Fig. 3d) neurons, the current-voltage relationship was found to be linear near the resting potential, in the range of −40 mV to −90 mV (Fig. 3c,d, right panels). The slope of the curve, corresponding to the DC conductance of the membrane, increased when the amplitude of the step potential exceeded −40 mV, which was most likely due to the activation of voltage-gated channels (Fig. 3c,d, left panels). This range, however, is beyond what is analysed in the current manuscript.

We further analysed the voltage-dependence of the membrane impedance by comparing impedance magnitudes, |*Z*_m_|, at various *V*_m_ in the range of −40 mV to −90 mV. As summarized in Fig. 3e, the low-pass filtering property in the range of 10 Hz to 90 Hz was unaffected by *V*_m_. In contrast, |*Z*_m_| at each frequency was found to increase with *V*_m_ indicating a contribution of voltage-gated conductances in determining |*Z*_m_|. This trend is consistent with previous literature on recordings of pyramidal neurons in hippocampal slice preparations^15^. This increase in |*Z*_m_| with *V*_m_ abolished when voltage-gated K^+^ channels were blocked by tetraethylammonium (TEA; 14 mM) (Fig. 3f). At *V*_m_ = −70 mV, however, the effect of TEA on |*Z*_m_| was insignificant at all frequencies. The latter result suggests that the voltage-gated K^+^ conductance plays a minor role in determining *Z*_m_ near this membrane potential. In sum, the above experiments justify that the membrane property near *V*_m_ = −70 mV in the presence of TTX and CNQX is dominated by the passive membrane property.

Comparison of impedance spectrum in patterned and unpatterned neurons is summarized in Fig. 3g,h. Intriguingly, despite the morphological confinement that reduced the total area of the cell membrane, the difference in |*Z*_m_*|* of patterned and unpatterned neurons were statistically insignificant for the case of 7 DIV neurons (Fig. 3g). This effect was also observed even in the presence of TEA, suggesting that the effect is independent of voltage-gated K^+^ conductance (Fig. 3g, inset). The contribution of HCN channels should be minor since *Z*_m_ was measured at *V*_m_ = −70 mV^9,15^. The influence of voltage-gated Ca^2+^ currents should also be minor since it is negligibly small at *V*_m_ < −30 mV^34^. At 16 DIV, |*Z*_m_*|* of the unpatterned neurons was found to be statistically lower than that of the patterned neurons at 10–50 Hz, but the decrease was limited to merely ~30% (Fig. 3h).

The phase angle of *Z*_m_ for the patterned and unpatterned neurons are shown in the lower panels of Fig. 3g,h. The negative phase shift indicates that the impedance is capacitive. The result is in agreement with previous electrophysiological recordings of hippocampal pyramidal neurons^9^ and computational modelling of passive neurons^12,35^. No significant difference in the phase profile was observed between the patterned and unpatterned cultures at both 7 and 16 DIV.

### Simulation results

**Figure 4:**
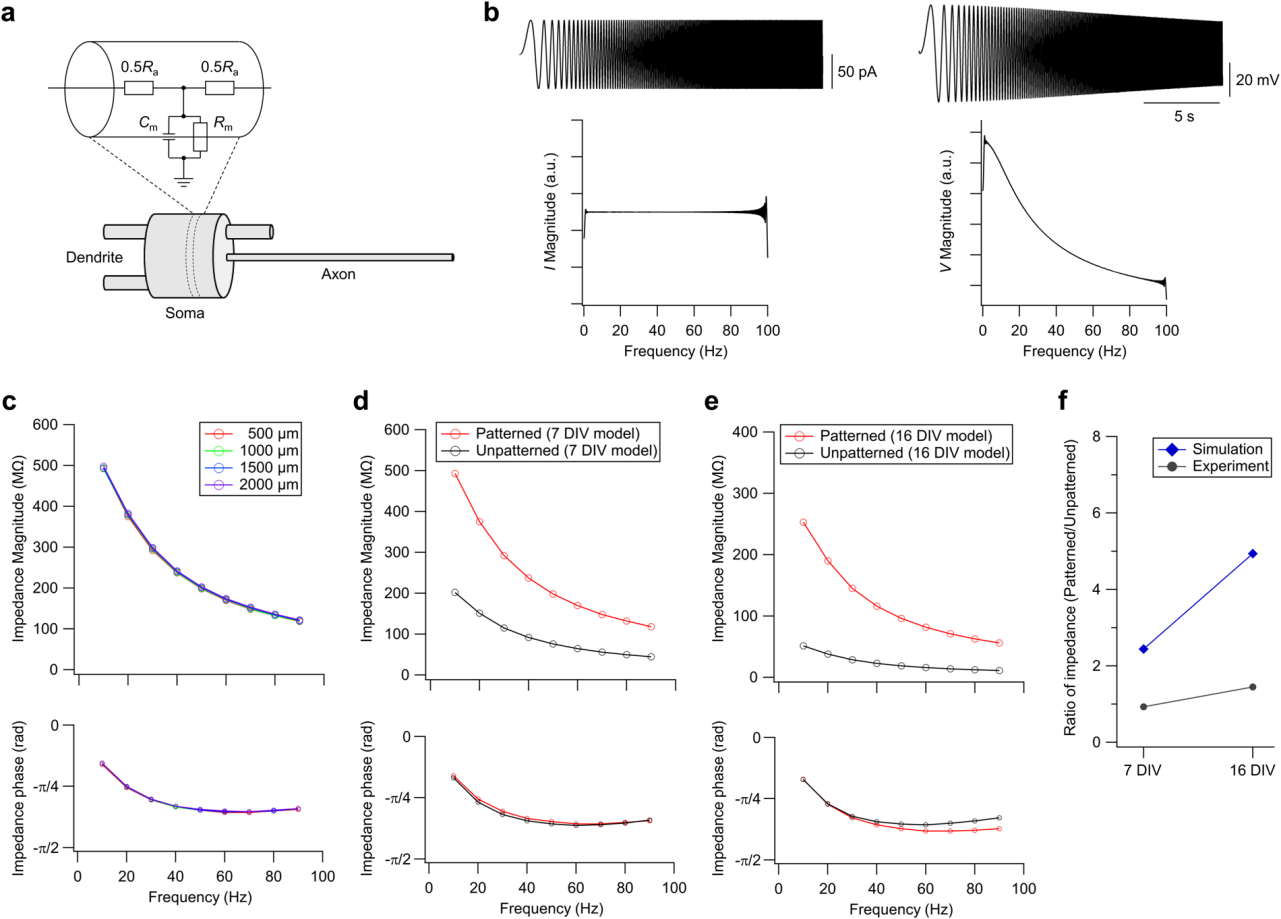
Computational modelling of the effect of cell morphology on membrane impedance. (**a**) Schematic representation of the model. (**b**) Traces (top) and their magnitude spectra (bottom) of the applied chirp current (left) and the voltage response (right) from the 7 DIV patterned model. Only the first 20 s are shown in the traces. (**c**) Comparison neuron models with different axon length. Using the patterned neuron model at 7 DIV, axon length was varied within the range of 500–2000 μm. Upper and lower panels show the impedance magnitude and phase profiles, respectively. (**d,e**) Comparison of patterned (red) and unpatterned (black) neuron models which were constructed to mimic the morphology of biological neurons at (**d**) 7 DIV and (**e**) 16 DIV. Upper and lower panels show the impedance magnitude and phase profiles, respectively. (**f**) Ratio of the membrane impedance at 10 Hz in patterned and unpatterned neurons.

In order to theoretically investigate the effect of cell morphology on *Z*_m_, we prepared multi-compartmental models for the patterned and unpatterned neurons at 7 and 16 DIV (Fig. 4a,b). A representative neuron with a morphology closest to the average values was selected and used as the model for each experimental condition (see Methods and Table 1). Since the voltage-gated Na^+^ channel and the glutamatergic receptor were pharmacologically blocked in experiments, only the passive properties, which include the specific membrane resistance (*R*_m_ = 10 kΩ cm^2^), specific membrane capacitance (*C*_m_ = 1 μF cm^−2^), and specific axial resistance (*R*_a_ = 150 Ω cm), were inserted in the model membrane. Due to its small diameter that increases the axial resistance, axon length does not substantially influence *Z*_m_ (Fig. 4c), as compared to dendrites. Therefore, we set the axon length to a constant value for all models and focused on the effect of soma and dendrite morphology.

**Table 1:** Summary of dendrite lengths, *L*, and their connection sites of the simulation models.

Dend #	*L* (μm)	Connected to:	Dend #	*L* (μm)	Connected to:
*7 DIV patterned model*			*16 DIV unpatterned model*		
D1	22.3	Soma	D1	66.7	Soma
D2	25.8	Soma	D2	154.8	Soma
D3	19.7	Soma	D3	129.2	Soma
			D4	162.7	Soma
*7 DIV unpatterned model*			D5	138.3	Soma
D1	133.6	Soma	D6	200.9	Soma
D2	8.0	Soma	D7	76.6	Soma
D3	137.5	Soma	D8	124.8	Soma
D4	25.6	Soma	D9	79.5	Soma
D5	24.5	Soma	D10	59.4	D2
D6	7.3	D1	D11	55.6	D2
D7	6.3	D1	D12	37.6	D4
D8	5.8	D1	D13	28.9	D4
D9	44.4	D3	D14	54.0	D5
			D15	72.4	D5
*16 DIV patterned model*			D16	79.5	D6
D1	97.2	Soma	D17	33.8	D6
D2	11.9	Soma	D18	46.4	D8
			D19	40.7	D8
			D20	40.9	D16
			D21	29.7	D20
			D22	18.7	D18

Figure 4d,e show the frequency response curves obtained from the patterned and unpatterned neuron models. The low-pass characteristics that were observed in the patch-clamp experiments were also confirmed in the simulations. This indicates that the spectral properties are supported by the passive property of the neuronal cell membrane, which can be equivalently modelled with an RC parallel circuit.

Notably, although the general trend was consistent, the change in |*Z*_m_*|* due to morphological confinement was quantitatively different in the experimental data and the simulation. The ratio of |*Z*_m_*|* of patterned and unpatterned neurons are summarized in Fig. 4f. Since the specific resistance of the cell membrane assumed in the model directly influences |*Z*_m_*|*, we evaluated the effect of geometrical confinement based on the ratio at 10 Hz. In biological neurons, morphological confinement had no significant effect on |*Z*_m_*|* in the 7 DIV neurons and caused 1.5-fold increase in the 16 DIV neurons. To the contrary, morphological confinement resulted in 2.4- and 4.9-fold increase in the 7 and 16 DIV neuron models, respectively.

This result suggests that in biological neurons, *Z*_m_ is homeostatically regulated to impede a minor perturbation in cell morphology. When the perturbation is substantially large, e.g., in the case of patterned and unpatterned neurons at 16 DIV, the size effect exceeds the regulatable range, and |*Z*_m_*|* is subjected to change (see Fig. 3h).

### Functional role of neuronal membrane impedance

**Figure 5:**
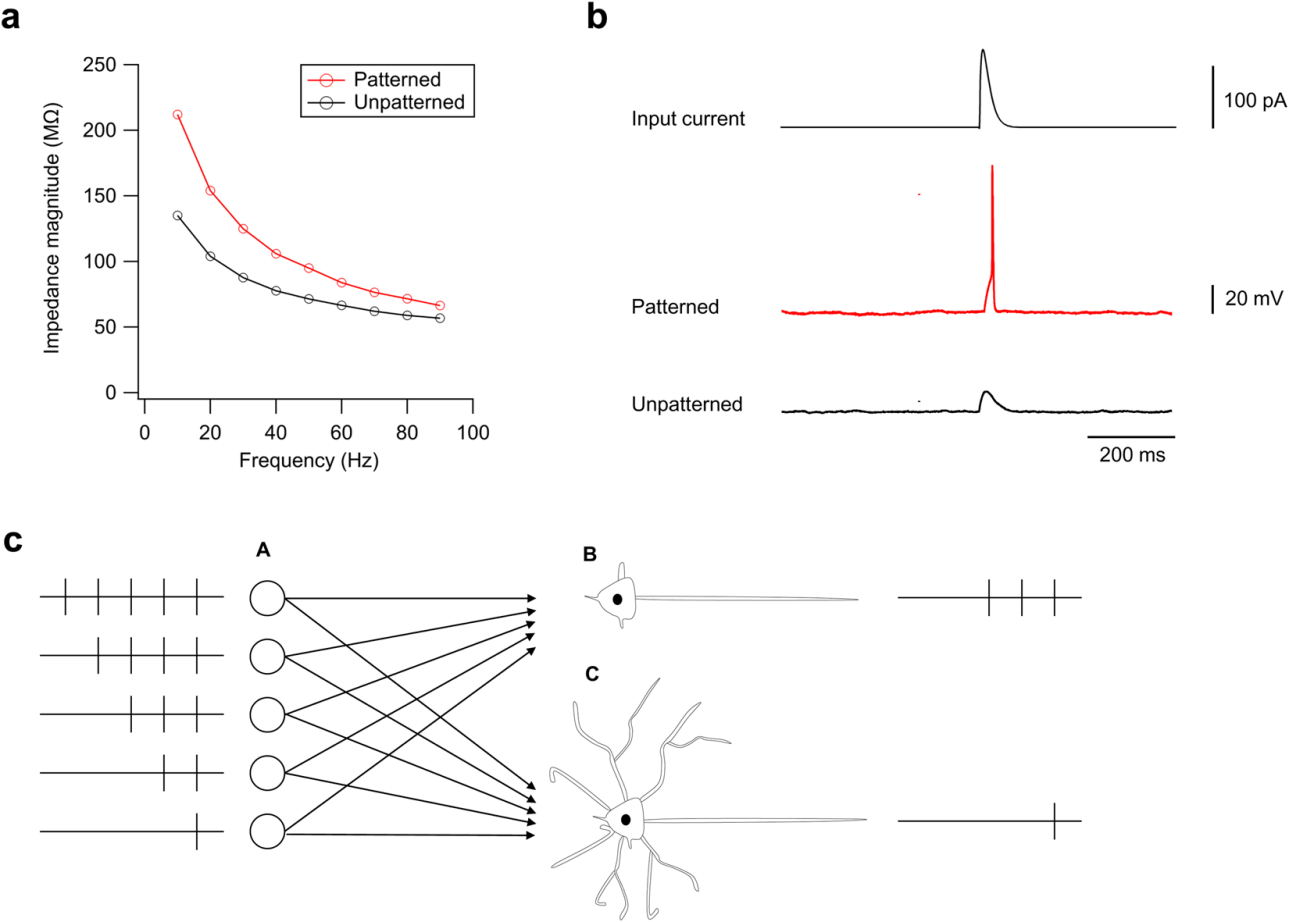
Functional role of membrane impedance. (**a**) Impedance magnitude profiles of patterned (red) and unpatterned (black) neurons at 16 DIV. (**b**) Voltage responses of the same neurons whose impedance profiles were presented in (**a**). The same input was applied to both neurons. (**c**) A schematic illustration of the effect of the membrane impedance on the coincidence detection property: Input neurons (A) send different pulse trains to a high-impedance (B) and low-impedance (C) neurons. Neuron B requires only three coincident inputs to fire, while Neuron C requires five coincident inputs. Although the influence of individual synapses is smaller in Neuron C, the larger dendritic field would permit the neuron to receive synaptic inputs from more neurons than Neuron B.

Finally, we demonstrate how the difference in *Z*_m_ can affect the function of a neuron in a network. Here, |*Z*_m_| of two neurons at 16 DIV were measured in advance (Fig. 5a), and a current which mimicked the excitatory post-synaptic current was then applied to the same neurons. When an ionic or electrical current *I* is delivered to a neuron, the change in *V*_m_ is given by *Z*_m_·*I*. Therefore, the amount of inputs necessary for *V*_m_ to increase above the threshold voltage and the neuron to fire an action potential is critically dependent on *Z*_m_.

A comparison of the patterned and unpatterned neurons revealed that the voltage response of the patterned neuron was larger for an identical input. In the case shown in Fig. 5b, the input triggered an action potential in the patterned (high-impedance) neuron, whereas this was not observed in the unpatterned (low-impedance) neuron. Since neurons are threshold units, a minor difference in the voltage response substantially affects the neuron’s behaviour, especially at *V*_m_ near the threshold. From the viewpoint of neural computation, the values of neuronal *Z*_m_ thus determine the number of synaptic inputs necessary for a neuron to send an output signal. Considering that low-impedance neurons have a large dendritic field, such neuron is electrically and morphologically optimized as an integrator of a large number of inputs (Fig. 5c). In contrast, high-impedance neurons would be an optimal relay element that sends output responding to a small number of inputs.

## Discussion

In this study, we employed micropatterned substrates to geometrically confine cultured hippocampal neurons and studied the effect of morphological constraint on *Z*_m_ that determines the input-output relation of neurons. Comparison of the electrophysiological data obtained from cultured hippocampal neurons with multi-compartmental neuron models showed that *Z*_m_ of biological neurons is homeostatically regulated to impede minor perturbations in cell morphology.

The molecular mechanism behind the homeostatic regulation of *Z*_m_ remains to be solved. Since the experiment was performed under activity-independent conditions and that homeostasis was observed on different culture days, a persistent change in the cell membrane, such as a change in leak-channel conductance, should underlie this phenomenon. One possible mechanism is that the density of leak channels is increased in the case of the confined culture. Another possible factor is the influence of mechano-gated K^+^ channels^36–39^. TREK-1 (TWIK-related K^+^ channel) channel, which belongs to a subclass of two-pore-domain of K^+^ channels, is extensively expressed in the entire cell membrane of hippocampal pyramidal neurons^39^. Morphological confinement could induce a constant stress on the cell membrane and increase the open probability of the TREK-1 channel, which results in a decrease in |*Z*_m_|. Lipid composition affects specific capacitance of bilayer lipid membranes^40^, which can result in a perturbation of |*Z*_m_|, but the range of variation in specific capacitance is rather small to describe the homeostatic regulation solely on this mechanism.

Under a physiological condition, the passive (low-pass) membrane property interacts with active conductance governed by voltage-gated ion channels, such as the HCN channel^6–14^ and the M-type K^+^ channel^10,15^, which act as high-pass filters. In subpopulations of neurons, such as the regular spiking and intrinsic bursting neurons in the neocortex^2^, stellate cells in the entorhinal cortex^16,17^, and a small fraction (~20%) of hippocampal pyramidal neurons^15^, the high-pass filtering property is significantly strong, and the neuronal membrane is endowed with a band-pass filtering property that regulate rhythmic activity in the neuronal network. The passive membrane impedance also influences the function of such band-pass neurons, thus the mechanism presented here represents a potentially general strategy for tuning the network function and dynamics in the nervous system.

In summary, we provided direct evidence that biological neurons bear a cell-autonomous mechanism for homeostatically retaining *Z*_m_ when cell morphology is subjected to a minor deviation. We also found that |*Z*_m_| changes in a size-dependent manner when the deviation is large. This influences the neuron’s function as a coincidence detector, or the “quorum” of neuronal firing. Since this property strongly affects the spontaneous activity patterns^41,42^ and noise-sensitivity of neuronal networks, it would be of great interest to examine the influence *Z*_m_ modification on the spatiotemporal patterns of network activity. Development of a patterning method that permits a graded control of dendritic lengths and soma sizes would assist further research in this direction, as well as in elucidating the underlying mechanism of *Z*_m_ homeostasis. Not only during development but also in mature brains, neurons are subjected to a constant perturbation in membrane area. The homeostatic mechanism presented in this study would help neuronal networks to operate resiliently under such perturbations.

## Methods

### Microcontact printing

The procedure of microcontact printing used to fabricate the patterned substrate has been described previously^31^. Briefly, glass coverslips were fully cleaned by ultrasonication in 100% ethanol for 5 min, rinsed three times in Milli-Q grade water, and treated in an air-plasma for 60 s (Yamato PM100). Several dots of paraffin wax (Sigma P3558) were then placed at the periphery of the coverslip to provide a ~0.5 mm spacing between the coverslip and glial feeder layers during cell culturing. The substrate was then coated with 0.2% agarose (Sigma A9918), dried overnight, and sterilized under UV light for 30 min. PDMS stamps were cleaned by ultrasonication in 100% ethanol for 5 min, dried in air, and the surface of the stamps were then coated with a protein ink solution. The ink solution consisted of 50 μg mL^−1^ poly-D-lysine hydrobromide (Sigma P0899) and extracellular matrix gel (Sigma E1270; 1:100 dilution) in Dulbecco’s modified Eagle’s Medium (Gibco 10569-010). After drying the protein ink, the PDMS stamps was pressed against the agarose-coated glass coverslips using a single-axis micromanipulator.

### Cell culture

All experiments were approved by the Center for Laboratory Animal Research at Tohoku University and were performed in accordance with the University’s guidelines. Timed-pregnant Sprague Dawley rats were obtained from Charles River Laboratories Japan, and hippocampal neurons were dissociated from embryonic day 18 pups. The neurons were plated on the patterned glass coverslips at a density of 7.0 × 10^3^ cells cm^−2^ in the neuronal plating medium [5% fetal bovine serum (Gibco 16140-071) and 0.55% glucose in minimum essential medium (MEM; Gibco 11095-080)]. After 3 h, the coverslip was flipped upside-down and transferred to a 60-mm dish with an astrocyte feeder layer containing 4 mL of N-2 culture medium [10% N2 supplement, 0.5 mg mL^−1^ ovalbumin (Sigma A2512), and 10 mM HEPES in MEM]. After 4 DIV, 1 mL of Neurobasal medium [2% B-27 supplement (Gibco 17504-044) and 1% GlutaMAX-I (Gibco 35050-061) in Neurobasal medium (Gibco 21103-049) containing 1 μM cytosine arabinoside] was added to each culture dish. At 8 DIV, 2 mL of the culture medium was replaced with a conditioned Neurobasal medium.

### Immunocytochemistry

Neurons were fixed in 4% paraformaldehyde/4% sucrose solution for 15 min at 37 °C, permeabilized with 0.25% Triton X-100, and blocked with 0.5% fish skin gelatin at 7 and 16 DIV^26^. The following antibodies were used: anti-SMI312 (BioLegend 837904, mouse IgG1/IgM, 0.5 μg mL^−1^), anti-MAP2 [Abcam ab5392, chicken polyclonal IgY (IgG), 2.5 μg mL^−1^], Alexa 488-labeled goat anti-mouse IgG1 (Molecular Probes A21121, 2 μg mL^−1^), and Alexa 568-labeled goat anti-chicken IgG (Molecular Probes A11041, 8 μg mL^−1^).

### Electrophysiology

Whole-cell patch-clamp recordings (HEKA EPC-10) were performed at 7 and 16 DIV. The extracellular solution for the recording contained (in mM): 140 NaCl, 2.4 KCl, 10 HEPES, 10 glucose, 2 CaCl_2_, 1 MgCl_2_ (pH 7.4). To block any voltage-dependent Na^+^ currents and glutamatergic neurotransmission, 1 μM TTX (Latoxan L8503) and 10 μM CNQX (Sigma C127) were added to the solution. In some experiments, 14 mM TEA (Wako 206-04501) was additionally included in the extracellular solution to block voltage-dependent K^+^ currents. Borosilicate micropipettes (Sutter BF 150-86-10) were pulled by a micropipette puller (Sutter P-97) immediately before each recording. The intracellular solution contained (in mM): 146.3 KCl, 0.6 MgCl_2_, 4 ATP-Mg, 0.3 GTP-Na, 5 U mL^−1^ creatine phosphokinase, 12 phosphocreatine, 1 EGTA, 17.8 HEPES (pH 7.4). Signals were sampled at 20 kHz and filtered with 10 kHz and 2.9 kHz Bessel filters. Recordings were performed at room temperature.

The current-voltage relationship of neurons were obtained by first clamping *V*_m_ at −70 mV and then applying test pulses of −90 mV to +40 mV (duration, 500 ms). The mean evoked current of the last 50 ms, offset by the membrane current before stimulation, was analysed. To obtain membrane impedance spectra, a sinusoidal current, *i*_in_, with a constant amplitude of 50 pA, a linear or exponential frequency span of 0.1–100 Hz, and a duration of 50 s was applied to neurons under the current-clamp mode. Precisely, *i*_in_ of a linear chirp input is given by:

1 $${i}_{{\rm{in}}}=a\,\sin [2\pi ({f}_{0}+\frac{k}{2}t)t],$$

where *a* = 50 pA is the amplitude, and $k=({f}_{1}-{f}_{0})/T$ is the rate of frequency increase with *f*_0_ = 0.1 Hz, *f*_1_ = 100 Hz, and *T* = 50 s. Correspondingly, *i*_in_ of an exponential chirp input is given by:

2 $${i}_{{\rm{i}}{\rm{n}}}=a\,\sin [\frac{2\pi {f}_{0}}{{\rm{l}}{\rm{n}}(k)}({k}^{t}-1)].$$

Unless otherwise noted, the membrane potential was set to −70 mV with current injection. The discrete Fourier transforms of *i*_in_ and the voltage response (*v*_out_), which we denote *I* and *V*, respectively, were then obtained using the FFT function implemented in Python-NumPy. The magnitude and argument (or phase) of *I* and *V* were then:

3 $$|X|=\sqrt{X\cdot {X}^{\ast }},$$

4 $$\text{arg}(X)={\tan }^{-1}(\frac{{\rm{Im}}[X]}{{\rm{Re}}[X]}),$$

where *X* is either *I* or *V*, $|\,\cdot \,|$ the magnitude, * the complex conjugate, arg$(\,\cdot \,)$ the argument, and Re[*X*] and Im[*X*] are the real and imaginary parts of *X*, respectively. Finally, the magnitude and the phase of impedance (*Z*_m_) were derived by:

5 $$|{Z}_{{\rm{m}}}|=\frac{|V|}{|I|},$$

6 $$\text{arg}({Z}_{{\rm{m}}})=\text{arg}(V)-\text{arg}(I).$$

The data are presented by taking an arithmetic average over ± 0.2 Hz around 10, 20, …, 90 Hz. Values in the range of 50 ± 0.1 Hz were eliminated from the analysis in order to filter out possible AC power noise.

For applying synaptic current to neurons, the current-clamp amplifier was driven by an analogue signal from a computer running the Real-Time Experimental Interface^43^. The synaptic current at time *t*, *i*_syn_(*t*), was calculated from the following equation:

7 $${i}_{{\rm{syn}}}(t)={g}_{{\rm{syn}}}(t)\cdot [{V}_{{\rm{m}}}(t)-{E}_{{\rm{syn}}}],$$

8 $${g}_{{\rm{s}}{\rm{y}}{\rm{n}}}(t)={g}_{max}\cdot \frac{t}{\tau }\cdot \exp (-\frac{t-\tau }{\tau }),$$

where *g*_syn_ is the synaptic conductance, *V*_m_ the membrane potential of the recorded neuron, *E*_syn_ the reversal potential of an excitatory synapse (20 mV), ${g}_{{\rm{\max }}}$ the maximum synaptic conductance (20 nS) and *τ* the membrane time constant (10 ms).

### Simulation

The neuron model was implemented in the NEURON software^44^ and was used to theoretically investigate the morphology-dependence of *Z*_m_. A multi-compartmental neuron model consisting of a soma attached to an axon and dendrites were prepared. The models of the patterned and unpatterned neurons at 7 and 16 DIV were constructed to mimic the soma and dendritic morphology of biological neurons, which were determined by tracing the immunostained sample. Soma was represented with a cylinder having identical values of diameter and height, which were 16.9 μm (7 DIV patterned), 20.9 μm (7 DIV unpatterned), 26.7 μm (16 DIV patterned), and 42.5 μm (16 DIV unpatterned). Dendrites were represented with 2 μm-diameter cylinders with varying lengths as summarized in Table 1. For modelling axons, the length of cylindrical compartment was kept constant to be 500 μm for all neuron models unless otherwise noted, and the diameter was set to be 0.5 μm. The specific membrane capacitance, specific membrane resistivity, specific axial resistance were set to be 1 μF cm^−2^, 10 kΩ cm^2^, and 150 Ω cm, respectively. The values of the parameters were based on previous literature reports on the hippocampal neuron model^11,45^. A chirp current with an amplitude of 50 pA, a linear frequency span of 0.1–100 Hz, and a duration of 100 s was injected to the models, and the impedance spectra was obtained as described in the previous section.

### Data availability

The datasets generated and analysed during the current study are available from the corresponding author on reasonable request.
